# A Simple Model to Predict the Probability of a Peach (*Prunus persicae*) Tree Bud to Develop as a Long or Short Shoot as a Consequence of Winter Pruning Intensity and Previous Year Growth

**DOI:** 10.1371/journal.pone.0052185

**Published:** 2012-12-26

**Authors:** Daniele Bevacqua, Michel Génard, Françoise Lescourret

**Affiliations:** INRA, UR1115 PSH, Avignon, France; United States Department of Agriculture, United States of America

## Abstract

In many woody plants, shoots emerging from buds can develop as short or long shoots. The probability of a bud to develop as a long or short shoot relies upon genetic, environmental and management factors and controlling it is an important issue in commercial orchard. We use peach (*Prunus persicae*) trees, subjected to different winter pruning levels and monitored for two years, to develop and calibrate a model linking the probability of a bud to develop as a long shoot to winter pruning intensity and previous year vegetative growth. Eventually we show how our model can be used to adjust pruning intensity to obtain a desired proportion of long and short shoots.

## Introduction

Two morphologically distinct shoots, commonly referred to as short and long shoots (SS and LS, respectively), occur in many woody plants. In SS the rib meristems fails to become active after opening of the buds so that little or no intermodal elongation occurs. The putative long and short shoots buds are generally identical and differences emerge during growing season. Both type of shoots bear foliage and contribute to photosynthesis. Short shoots generally do not exceed 2 cm length and are important providers of photosynthate in the first weeks following bud breaking, whereas LSs have elongated stems and constitute tree architecture [1]. The probability of a bud to develop as LS (P_LS_) is controlled by both genetic, environmental and, when present, management factors. For example in genera *Pinus* and *Larix* it is almost constant. A wider range in the proportion between SSs and LSs seems to exist in deciduous rather than coniferous trees. In some genera (*e*.*g*. *Fagus*, *Betula* and *Acer*) it is less predictable than in others (*e*.*g*. *Malus*, *Prunus*, *Pyrus*, *Cytrus*, *Cratageus etc*.), although P_LS_ generally decreases with age.

In commercial orchards, the quantity of 1-yr old LS in trees is artificially regulated by winter pruning intended to shape trees and adjust crop load in order to *i*) improve or maintaining tree vigor and *ii*) increase the yield or quality of fruits [2]. In fact, pruning alters the shoot : root ratio, by removing shoot biomass, and forces the plant to increase new shoot growth, according to the functional-balance concept [3] which states that new biomass is partitioned between roots and shoots in favor of the organ that capture the limiting resource (e.g. carbon or nitrogen, respectively captured by shoots and roots). In the growing season, new shoots will then emerge from remaining 1-yr old LSs (i.e. those LSs that have not been cut). Consequences of winter pruning on fruit production are not straightforward since it generally increases the fraction of buds that develop into LS, but it also eliminates 1-yr old shoots bearing flower and vegetative buds. Moreover, some trees bear most of fruits on SSs (e.g. cherry trees, apple trees) while others such as peach trees bear fruits on LSs [2].

Quantitative relationship between cultural practices and shoots development should be explicitly considered in mathematical crop models. These models can then be used to predict crop dynamics under different cultural practices, for which direct field observations would be extremely difficult and/or expensive [4]. Despite winter pruning is one of the most common cultural practice influencing P_LS_, there are no dedicated studies to quantify its effect on P_LS_ (but see Grechi et al. [5] who studied the effect of winter pruning on peach tree-aphid interactions and also provided a first estimate of the effect of winter pruning on P_LS_). Bussi et al. [6] focused on probability of sprouts emergency as response to pruning intensity. Stephan et al. [7] analyzed the effect of pruning intensity on apple tree architecture and Gordon & Dejong [8] studied the effect of sprouts removal and fruit crop on P_LS._ Fumey et al. [9], in a comprehensive experimental study, analyzed the consequences of different pruning practices on tree branching in apple trees. They found that pruning enhanced vegetative growth and decreased flowering, yet they did not provide a quantitative model to predict the consequences of different practices. In present work we use peach (*Prunus persicae*) trees, subjected to different winter pruning levels, and monitored for two consecutive years, to develop and calibrate a model linking P_LS_ to intensity of winter pruning and overall length of 1-yr old shoots before pruning. A high capacity for neoformation determines high plastic adaptation in response to branch removal and makes peach a good model to study the effects of winter pruning [8]. We use the calibrated model to assess the number of long shoots N_LS_ relevant to different combinations of winter pruning intensities and 1-yr old wood present before pruning. Non-linearity of interactions between considered variables gives rise to a maximum value of N_LS_ at different values of pruning intensity, depending on the overall length of 1-yr old shoots before pruning. Eventually, we show how our model can be used to adjust winter pruning intensity to get an optimal production of LSs.

## Materials and Methods

### Available Data

Data were collected in 2005 and 2006 from an experimental peach orchard planted in 1998 with 20 late maturing trees (cv Suncrest/GF677) (see Grechi et al. [5] for a full description of the experiment). Trees were pruned in winter with a pruning intensity (PI) (i.e. fraction of mass of 1-yr old wood pruned on total 1-yr old wood) varying from 0 to 0.8. Each year, for each tree, we measured tree length of 1-yr old wood before pruning (L_W1_), PI and the fraction of shoots that developed as long shoots (i.e. P_LS_). Data are reported in table 1.

**Table 1:** Characterization of the 20 peach trees monitored in 2005 and 2006: total length of 1-yr old wood before winter pruning (L_W1_), fraction of L_W1_ pruned (PI) and fraction of buds developing as long shoots (P_LS_).

Treeno.	L_W1_ (m)	PI	P_LS_	L_W1_ (m)	PI	P_LS_
	2005			2006		
1	190	0.4	0.9	157	0.11	0.14
2	170	0.24	0.23	152	0.36	0.14
3	164	0.56	0.23	247	0.63	0.36
4	182	0.39	0.25	309	0.49	0.42
5	185	0.32	0.9	259	0.49	0.24
6	158	0.49	0.25	250	0.56	0.36
7	138	0.5	0.13	131	0.14	0.9
8	215	0.28	0.8	143	0.36	0.25
9	230	0.64	0.36	354	0.66	0.52
10	186	0.65	0.36	357	0.77	0.50
11	265	0.48	0.20	184	0.54	0.34
12	269	0.15	0.16	106	0.21	0.22
13	265	0.65	0.23	190	0.68	0.61
14	219	0.61	0.24	182	0.73	0.50
15	298	0.26	0.7	101	0.38	0.17
16	264	0.34	0.9	137	0.46	0.24
17	220	0.39	0.16	178	0.49	0.23
18	282	0.19	0.11	170	0.34	0.24
19	226	0.54	0.19	157	0.56	0.41
20	222	0.10	0.8	144	0.25	0.18

No specific permits were required for the described field studies. The location is part of our public institute (INRA) domain. The field studies did not involve endangered or protected species.

### The Models

Being P_LS_ a probability (i.e. it ranges between 0–1) and assuming that its variability can depend on PI, L_W1_ and their interaction PI×L_W1_ (abundance of pruned 1-yr old wood), the full model is:
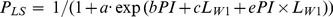
(1)where 1/(1+*a*) represents a possible constant value for P_LS_ and *b*, *c*, *e* are coefficients respectively accounting for the effect of winter pruning intensity, vegetative growth of last year and amount of pruned biomass.

To estimate the unknown parameters *a*, *b*, *c* and *e*, we linearized the model as follow:
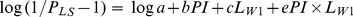
(2)


We selected the model providing the best fit to observed data through a backward stepwise selection procedure based on the Akaike information criterion (AIC).

After having checked for constancy of variance and normality of errors of the selected model, we estimated uncertainty associated with parameters of the best model by bootstrapping [10]. We resampled 10,000 times the original data, generating empirical probability distribution for each parameter.

We computed N_LS_ on a virtual tree, where L_W1_ varied between 0–600 m, and subjected to PI between 0–1, as:
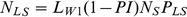
(3)where *N*_s_ is the constant number of shoots emerging per unit of 1-yr old wood left after pruning (N_S_ = 45.55 m^−1^ according to Grechi et al. [11]). Finally, we searched for the minimum value of PI that maximizes N_LS_, given different values of L_W1_. This is equivalent of finding the optimal level of PI in the case of a farmer that wanted to maximize N_LS_ in each tree. This is reasonable in most commercial peach orchards being LSs the most fruitful shoots [12].

## Results and Discussion

The best model explained 68% of observed P_LS_ variability and included L_W1_ and interaction L_W1_×PI as explanatory variables, while it excluded the sole effect of PI. Estimated model parameters and their empirical distributions are reported in table 2. The derivate of 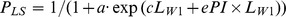 (i.e. the selected model to estimate P_LS_) with respect of PI is positive for 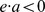 (see Supporting Information for details). Parameter estimates (*i*.*e*. *a* = 2.75 and *e* = −1.51×10^−2^) indicate thus that, in our considered model system, P_LS_ is a monotone increasing function of PI. In other words, the probability of a bud to develop into a LS is always increased by higher values of PI. On the other hand, the derivate of P_LS_ with respect of L_W1_ is positive for 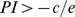 (see Supporting Information for details). Parameters estimates indicate thus that there is a critical value PI* = 0.52 over which trees with higher values of L_W1_ (referred to as “bigger trees” in the following) have higher P_LS_. Under this value of PI*, bigger trees are expected to have lower P_LS_. Note that L_W1_ is a proxy of previous tree growth and that with the term “bigger trees” we refer to those trees that produced more shoot biomass in the previous growing season and not necessarily to those that cumulated more biomass over the entire life span.

**Table 2:** Basic statistics of models parameters (see equations 3 and 4), as obtained by bootstrapping the 2005 and 2006.

Parameter	Mean	St.dev	Median	5^th^ percentile	95^th^ percentile
log *a*	1.01	0.25	1.02	0.59	1.41
*C*	7.85×10^−3^	1.83×10^−3^	7.78×10^−3^	4.83×10^−3^	10.8×10^−3^
*E*	−1.51×10^−2^	0.20×10^−2^	−1.51×10^−2^	−1.85×10^−2^	−1.2×10^−2^

**Figure 1:**
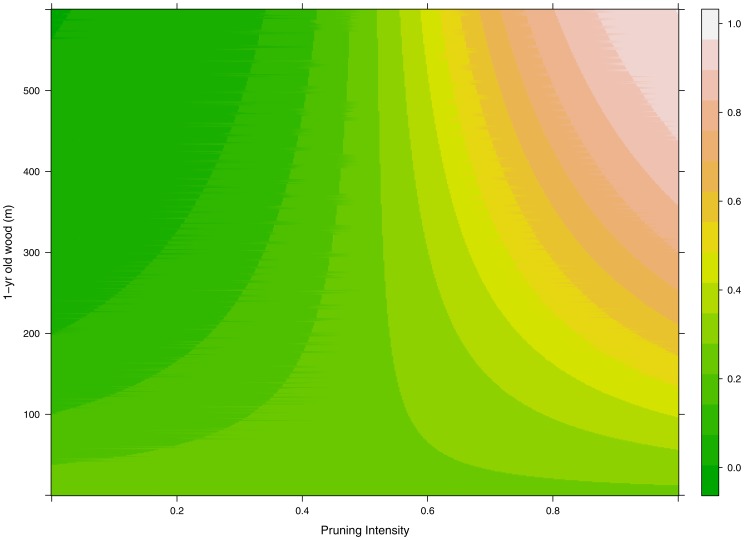
Estimated fraction of long shoots in the growing season (P_LS_) of a virtual peach *Prunus persica* tree as function of 1-yr old wood before winter pruning (L_w1_) and pruning intensity (PI, i.e. fraction of 1-yr old shoot removed before bud break).

**Figure 2:**
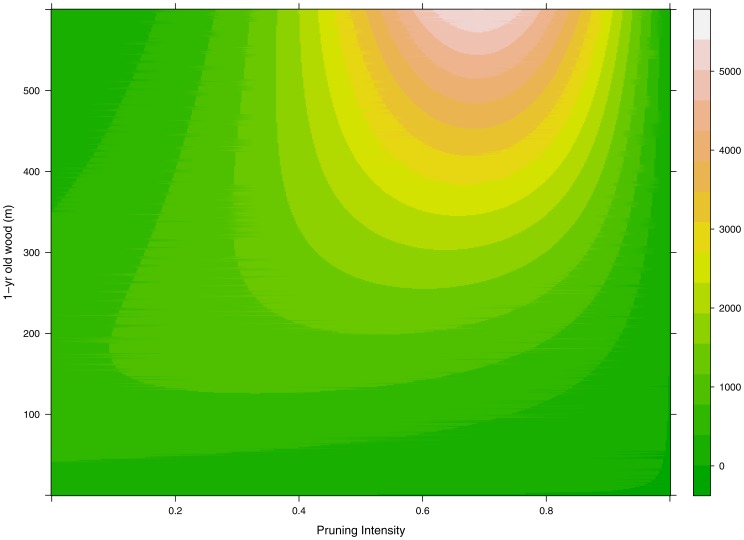
Estimated abundance of long shoots (N_LS_), in the growing season of a virtual peach *Prunus persica* tree as function of 1-yr old wood before winter pruning (L_w1_) and pruning intensity (PI,i.e. fraction of 1-yr old shoot removed before bud break).

**Figure 3:**
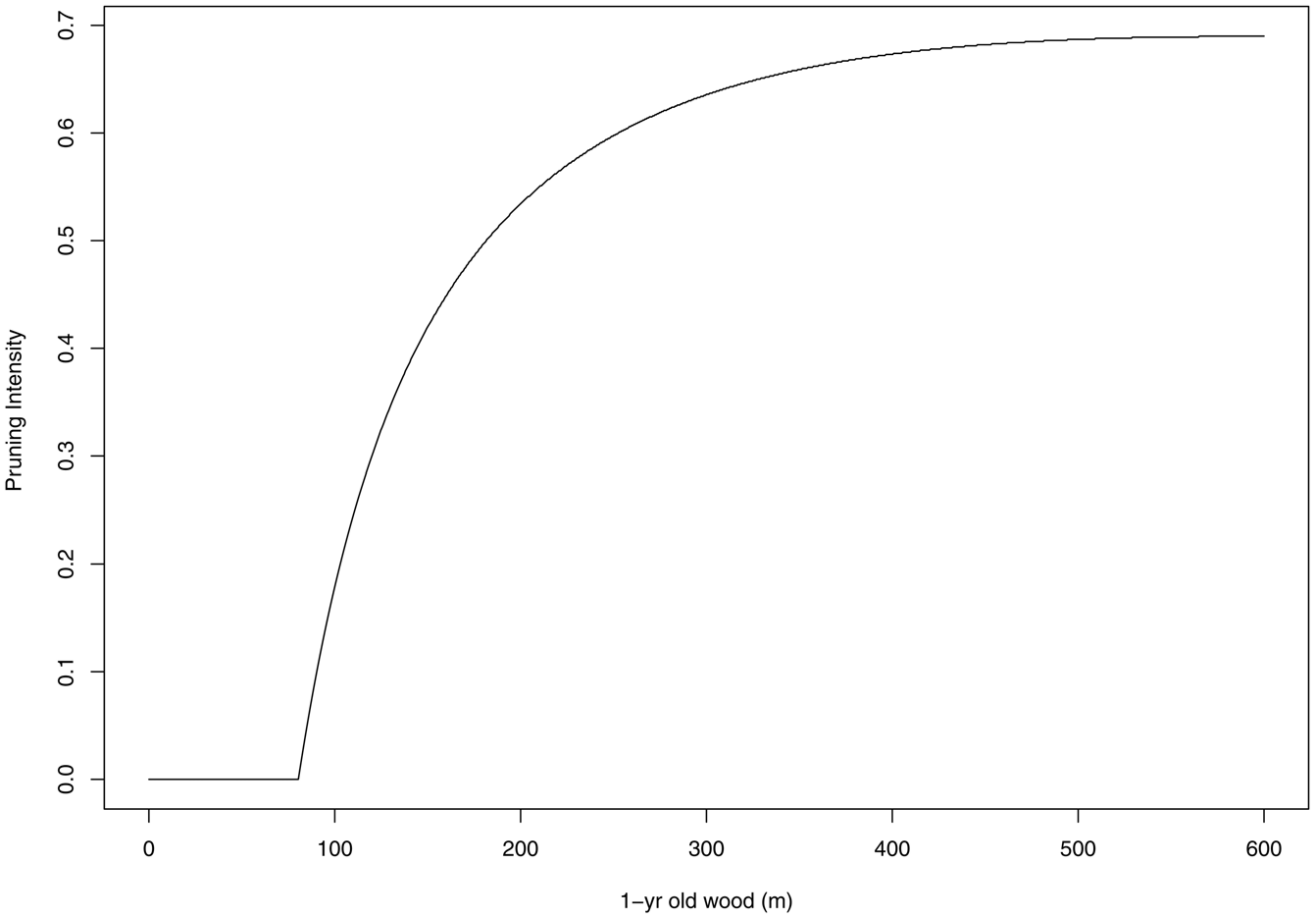
Pruning intensity (PI, i.e. ratio of 1-yr old shoot removed before bud break) determining the highest number of long shoots, as a function of 1-yr old wood before pruning (L_w1_).

The fraction of LSs and the overall number of LSs, predicted by the model for different scenarios of PI and L_W1_, are shown in fig. 1 and fig. 2 respectively. As discussed above, it is evident in fig. 1 that P_LS_ increases for increasing values of PI while the effect of L_W1_ depends on the PI level. According to our model, in order to maximize N_LS_, one should start to prune peach trees only when L_W1_>80 m and winter pruning should never cut more than 70% of 1-yr old wood (fig. 3). Figures of observed versus estimated values of P_LS_ and empirical parameters distributions are reported in figures S1 and S2.

The fact that P_LS_ increases with PI is coherent with previous studies (e.g. [5]) and with the functional balance theory [3]. Our results also suggest that, for low levels or absence of winter pruning (i.e. PI<0.52 in *P*. *persicae*), bigger trees produce a lower fraction of LSs. Similarly, Wilson [13] found that, on red maple (*Acer rubrum*) trees over 30-yr old, less than 10% of the shoots developed as LSs, and Greenwood et al. [14] found that old red spruce (*Picea rubens*) showed reduced LSs elongation. Such mechanism would allow the plant to maintain a fairly constant N_LS_ as bigger trees produce more shoots yet a lower fraction of long ones. Assuming N_LS_ as a proxy of peach tree vegetative growth potential in a growing year, our model suggests the existence of overcompensation in response to winter pruning only for plants with L_W1_>80 m. In fact, whenever winter pruning determines a higher N_LS_ with respect of undisturbed situation (i.e. PI = 0), it is the case of overcompensation i.e. the plant responds to a stress by increasing its ability to grow, and finally grows more than in undisturbed conditions [15]. Figure 3 shows that “disturbing” pruning practices become efficient only if L_W1_>80 m. Capacity for overcompensation in plants is likely to have evolved as a response to herbivory [16], it is more likely in environments with high nutrient availability [17], it is well documented in fruit trees ([18]; [19]; [20]) and it is well known by farmers that remove plant biomass with the final aim to increase shoot growth and related fruit production [2].

Farmer behavior in peach commercial orchards is well predicted by our simple model suggesting to exert low or no winter pruning intensity over small trees (i.e. trees with L_W1_<80 m, usually corresponding to trees <3-yr old) and gradually to increase pruning intensity until removing up to 70% of L_W1_ [21]. In late maturing peach orchards, increasing N_LS_ leads to an increase in fruit production. In fact, N_LS_ in a given year affects the number of fruits of the next year since peach flower buds and hence fruits are produced on 1-yr old long shoots. However fruit distribution on short and long shoots might highly vary between cultivars; other species bear fruits only on SS (e.g. cherry) with winter pruning increasing vegetative growth but decreasing yield in the following season [22] and other such as apple bear fruits on both LSs and SSs. Although farmer objective is likely to differ for different cultivars and species, our model would be useful to determine optimal winter pruning intensity according to different farmer objectives such as minimizing N_LS_ or obtaining an optimal ratio between LSs and SSs.

Grechi et al. [5], in a work focused on consequence of winter pruning on peach tree-aphid interactions, proposed an exponential relationship (i.e. 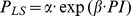) linking P_LS_ to the solely PI. Although that relationship highlighted the importance of PI on P_LS_, it can provide unrealistic biological results with P_LS_>1 and it neglects the previous growth of the tree. In the present work we overcome these main drawbacks since the image of function (1) ranges between 0–1 and the effect of previous growth on P_LS_ is considered through its proxy L_W1_.

We are conscious that a better insight into plant partitioning of new shoots in short and long ones will be achieved only by future experiments gathering information not just on plants having different value of L_W1_ and subjected to levels of winter PI, but also on plants having different ages and subjected to different pruning practices (e.g. summer *vs*. winter pruning, centrifugal *vs*. conventional etc.) and environmental stressors. More comprehensive dataset would also permit a validation of the model and possibly increase its predictive power. Yet, despite the above-mentioned limitations, our results are consistent with functional-balance theory and common cultivar practices, and the proposed model can help in modeling the effect of winter pruning above tree growth and fruit production.

## Supporting Information

Figure S1 **Estimated versus observed fraction of long shoots.**
(DOC)

Figure S2 **Variability of estimated parameters assessed via bootstrap (1000 iterations): bivariate scatter plots, linear fits and median values below and above the diagonal; histograms on the diagonal.** Pearson correlation are equal to −0.89 0.49 and −0.80 respectively between log *a*–*c*, *a*–*e*, and *c*–*e*.
(DOC)

Text S1 **Mathematical details.**
(DOC)
