# Supplementary figures and images for: A Simple Model to Predict the Probability of a Peach (Prunus persicae) Tree Bud to Develop as a Long or Short Shoot as a Consequence of Winter Pruning Intensity and Previous Year Growth
Source: PLoS One. 2012 Dec 26;7(12):e52185. doi: 10.1371/journal.pone.0052185 (PMC3530585; doi:10.1371/journal.pone.0052185)

SUPPORTING FIGURE S1


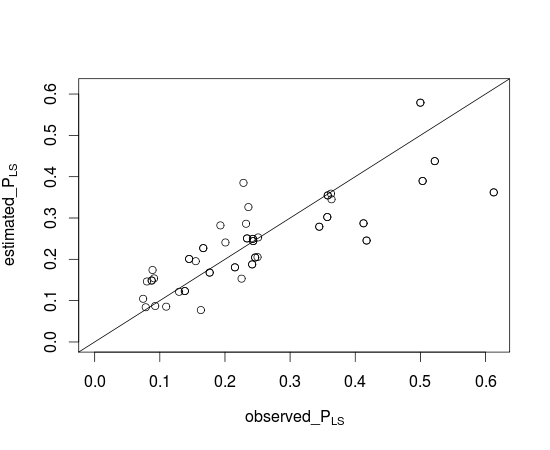

Supplement: Figure S1 — Estimated versus observed fraction of long shoots. (DOC) [file pone.0052185.s001.doc]

SUPPORTING FIGURE S2
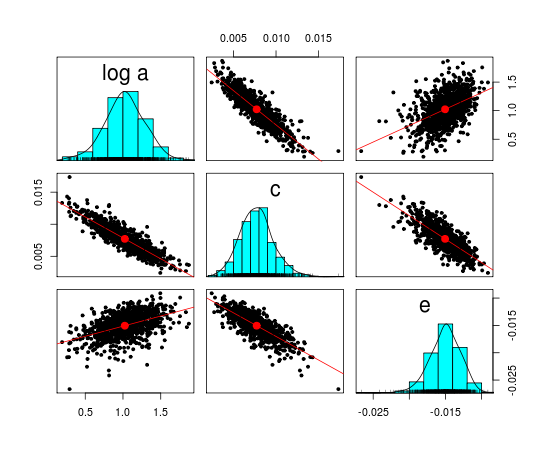

Supplement: Figure S2 — Variability of estimated parameters assessed via bootstrap (1000 iterations): bivariate scatter plots, linear fits and median values below and above the diagonal; histograms on the diagonal. Pearson correlation are equal to −0.89 0.49 and −0.80 respectively between log a–c, a–e, and c–e. (DOC) [file pone.0052185.s002.doc]
