# Supplementary material for: A Simple Model to Predict the Probability of a Peach (Prunus persicae) Tree Bud to Develop as a Long or Short Shoot as a Consequence of Winter Pruning Intensity and Previous Year Growth
Source: PLoS One. 2012 Dec 26;7(12):e52185. doi: 10.1371/journal.pone.0052185 (PMC3530585; doi:10.1371/journal.pone.0052185)
Supplement: Text S1 — Mathematical details. (DOC) [file pone.0052185.s003.doc]

MATHEMATICAL DETAILS

**The effect of PI on the estimated fraction of long shoots P_LS_**

$$\frac{dP_{LS}}{dPI}=-e\cdot a\cdot L_{W1}\cdot\exp(c\cdot L_{W1}+e\cdot L_{W1}\cdot PI)\cdot\left[1+\exp(c\cdot L_{W1}+e\cdot L_{W1}\cdot PI)\right]^{-2}$$

As being the product of three positive terms: $L_{W1}\cdot\exp(c\cdot L_{W1}+e\cdot L_{W1}\cdot PI)\cdot\left[1+\exp(c\cdot L_{W1}+e\cdot L_{W1}\cdot PI)\right]^{-2}>0$.

Then, $\frac{dP_{LS}}{dPI}>0$ when $-e\cdot a>0$

**The effect of L_W1_ on the estimated fraction of long shoots P_LS_**

$$\frac{dP_{LS}}{dL_{W1}}=-\left(c+e\cdot PI\right)\cdot a\cdot L_{W1}\cdot\exp(c\cdot L_{W1}+e\cdot L_{W1}\cdot PI)\cdot\left[1+\exp(c\cdot L_{W1}+e\cdot L_{W1}\cdot PI)\right]^{-2}$$

As being the product of three positive terms $L_{W1}\cdot\exp(c\cdot L_{W1}+e\cdot L_{W1}\cdot PI)\cdot\left[1+\exp(c\cdot L_{W1}+e\cdot L_{W1}\cdot PI)\right]^{-2}>0$.

Then, $\frac{dP_{LS}}{dL_{W1}}>0$ when $PI>-c/e$
